# Semi‐Telechelic Polymers from Mechanochemical C─C Bond Activation

**DOI:** 10.1002/advs.202304571

**Published:** 2023-10-23

**Authors:** Rony Schwarz, Charles E. Diesendruck

**Affiliations:** ^1^ Schulich Faculty of Chemistry and the Resnick Sustainability Center for Catalysis Technion – Israel Institute of Technology Haifa 3200008 Israel

**Keywords:** ball milling, c─c activation, doxorubicin, halogenation, mechanochemistry

## Abstract

Unstrained C─C bond activation is attained in homopolymers through mechanochemical bond scission followed by functionalization to yield mostly semi‐telechelic polymer chains. Ball milling poly(ethylene oxide) (PEO) in the presence of 1‐(bromoacetyl)pyrene (BAPy) yields the pyrene terminated PEO. Similarly, milling with 2,4′‐dibromoacetophenone followed by Suzuki coupling allows the introduction of various aryl end groups. PEOs with a molecular weight below 20 kDa show no functionalization, supporting a mechanochemical mechanism. The protocol is also tested with doxorubicin, yielding the drug‐polymer conjugate. PEO halogenation is also demonstrated by milling PEO with iodine, N‐bromosuccinimide, or N‐iodosuccinimide, which can then be reacted with an amine substituted anthracene. Grinding additional carbon polymers with BAPy indicates that this functionalization method is general for different polymer chemistries.

## Graphical abstract

Mechanochemical C─C bond activation is used to introduce different end groups to macromolecular chains, yielding mono‐telechelic polymers. Ball milling poly(ethylene oxide) in the presence of adequate small molecules enabled the functionalization with fluorescent tags, halogens, or a bromo‐aryl moiety, which undergoes further functionalization via Suzuki coupling. The protocol is demonstrated in several carbon‐based polymer chemistries.

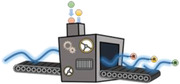

## 1 Introduction

**Scheme 1:**
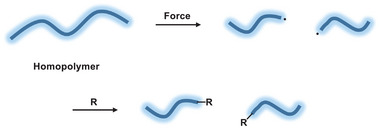
Functionalization of a homopolymer chain with an R group after its mechanochemical activation.

Selective activation of simple C*sp^3^*‐C*sp^3^* bonds is a highly desirable transformation in chemistry.^[^ ^1^ ^]^ Due to the high energy barrier for insertion into the C─C σ bond, this activation is challenging even when using highly reactive transition metal catalysts.^[^ ^2^ ^]^ Still, several examples of oxidative addition into three and four membered‐rings, or C─C bonds close to a directing group are found in the literature.^[^ ^3^, ^4^, ^5^ ^]^ Meanwhile, in polymer mechanochemistry, unstrained C─C bond scission is the most ubiquitous reaction, even in the absence of heteroatoms.^[^ ^6^ ^]^ Mechanochemical reactions require a minimal macromolecular length (typically presented in the form of molecular weight, *M*_lim_) and characteristically result in backbone scission and polymer length reduction, which is a main cause for the loss of polymers’ mechanical properties (mechanical aging).^[^ ^7^, ^8^ ^]^ This process was first studied by Staudinger in the early 1930s, when he observed the reduction in the viscosity over time of polystyrene during mastication.^[^ ^9^ ^]^ Staudinger's hypothesis of C─C bond cleavage in the polymer backbone was later supported by experimental studies by Sohma using electron spin resonance (ESR), confirming the formation of methylene radicals, which result from homolytic bond scission on the polymer's chain.^[^ ^10^ ^]^ While mechanochemical C─C bond activation occurs around the center of the chain (non‐selectively), in the early 2000s it was realized that selective cleavage of covalent bonds was possible by using strategically designed groups containing a mechanically weak chemical bond, named mechanophores.^[^ ^6^ ^]^ Mechanophores allowed the development of molecular‐level mechanoresponsive materials, leading to properties such as mechanochromism,^[^ ^11^, ^12^ ^]^ drug delivery,^[^ ^13^ ^]^ and intrinsic self‐healing.^[^ ^14^ ^]^ Yet, even in the absence of mechanophores, the commonly produced radicals were shown to induce chemical reactions. Upon C─C bond scission, the obtained macroradicals can act as initiators when in the presence of a monomer, yielding block co‐polymers.^[^ ^15^ ^]^ Kubota et al. demonstrated that a fluorophore connected to a nitroxide radical trap could be used to label polystyrene chains.^[^ ^16^ ^]^ More recently, Otsuka et al. created a molecular probe that catches macroradical through hydrogen transfer, creating a molecular fluorescent probe.^[^ ^17^ ^]^ Mechanically produced macroradicals were even shown to produce H_2_O_2_ in water.^[^ ^18^ ^]^ With multiple studies with and without mechanophores, the science and applications of polymer mechanochemistry have significantly advanced since its early days, providing new molecular approaches to mechanoresponsive materials,^[^ ^19^, ^20^, ^21^ ^]^ tools to study mechanics at the chemical level,^[^ ^22^, ^23^, ^24^ ^]^ and even affecting the pathway of organic reactions.^[^ ^25^, ^26^ ^]^ However the use of ball milling as a tool for the mechanochemically activation of C─C bonds^[^ ^27^ ^]^ followed by chemical reactions, is still very scarce. Ball milling was shown to be a useful tool for the solid‐state functionalization of small segments of poly(ethylene glycol) (PEG) by reacting the hydroxyl terminated groups in PEG chains with a base, followed by a reaction with an electrophile, but this did not involve a mechanical bond scission reaction.^[^ ^28^ ^]^ Here, we describe a first step toward the use of mechanical forces to induce unstrained C─C bond activation by introducing functional groups after the mechanically induced homolytic bond scission in homopolymers, leading to end‐functionalized (mostly semi‐telechelic) chains (**Scheme** **1**).

## 2 Results and Discussion

**Figure 1:**
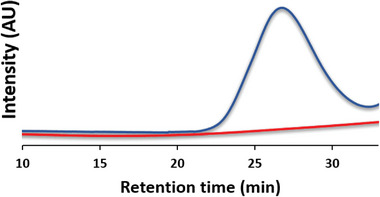
GPC chromatograms as observed by the UV detector at 356 nm of PEO 100 kDa after BMG with (blue) and without (red) BAPy.

Given that in most homopolymers mechanochemistry leads to C─C bond scission to form macroradicals, we hypothesized that grinding carbon‐based polymers in the presence of a small molecule capable of reacting with radicals would allow us to functionalize, at least in part, the product chains. Such molecules need to be relatively stable in the milling conditions but reactive toward relatively kinetically stable macroradicals.^[^ ^16^, ^29^, ^30^ ^]^ To test this hypothesis, poly(ethylene oxide) (PEO, Mw = 100 kDa) mixed with 1‐(bromoacetyl)pyrene (BAPy) was initially milled. PEO is expected to undergo a C─C bond scission, based on the work by Gaub et al., which simulated the chance of scission of unstrained C─C and C─O bonds.^[^ ^31^ ^]^ Such assessment was also supported by our own in silico simulations in which constrained geometries simulating external force (CoGEF) was done on triethylene glycol, leading to a C─C bond scission (see Section SIV, Supporting Information).^[^ ^32^, ^33^ ^]^ We hypothesized that the bromoacetyl moiety could serve as a good reaction site for a macroradical in a radical substitution reaction. Moreover, the pyrene moiety could serve as a marker identifiable by the UV–vis detector in the GPC (gel‐permeation chromatography). To our delight, ball‐mill grinding (BMG) such mixture under cryogenic conditions yielded the pyrene functionalized polymer as indicated by the characteristic absorption peak at 356 nm. BMG of the polymer without BAPy showed no polymer peak at 356 nm (**Figure** **1**). When the polymer is injected at a known concentration, the amount of chain functionalization could be calculated using the Beer‐Lambert law by integrating the GPC peak (see **Table** **1** and Section SIII, Supporting Information).

**Table 1:** BMG of PEO of different molecular weights with BAPy.^a)^

Entry	Initial Mw [kDa]^b)^	Final Mw [kDa]^c)^	Chains functionalized [%]^d)^
1	1.5	2.6 ± 1.3	0.0
2	4	4.9 ± 1.1	0.0
3	20	11.2 ± 1.1	2.7 ± 0.2
4	100	29.6 ± 1.7	36.2 ± 1.9
5	400	62.0 ± 11.5	45.3 ± 7.2
6^e)^	100	28.8 ± 0.7	75.6 ± 3.3

^a)^ The polymers were precooled to −196 °C using liquid nitrogen flow, then ball milled for three cycles of 10 min milling at 28 Hz and 2 min cooling at Hz. The values presented are an average of three independent BMG experiments;

^b)^ Mw as defined by supplier;

^c)^ Mw calculated using the GPC;

^d)^ (%) chains functionalized were calculated from the absorption of BAPy at 356 nm as described previously in the literature^[^ ^34^ ^]^;

^e)^ Six milling cycles were done instead of three.

**Scheme 2:**
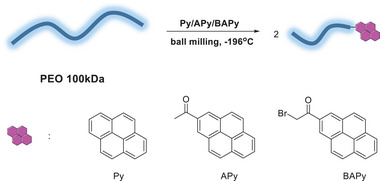
BMG of PEO 100 kDa at 28 Hz with Py, APy, or BAPy.

To test if the reaction is happening due to thermal shock or through mechanochemistry, PEOs with different molecular weights were subjected to BMG in the presence of BAPy. Results indicate that for polymers with a molecular weight below 20 kDa (≈*M*_lim_), no functionalization with BAPy was observed, supporting a pure mechanochemical mechanism (Table 1, entries 1 and 2). BMG of PEO 20 kDa yielded only mild functionalization (Table 1 entry 3), but grinding PEOs with higher molecular weights (entries 3–5), yielded increasing functionalization yields, typical of a mechanochemical process. Moreover, when grinding time was increased from 30 to 60 min, the amount of chain functionalization could be increased (Table 1 entries 4 vs. 6), reaching beyond 70% for PEO starting with 100 kDa (presuming one pyrene per chain). This pyrene functionalized polymer was further purified by dialysis, and analyzed by ^1^H‐NMR, in which aromatic peaks are seen between 8–9 ppm, at the same range as BAPy (see Figure S3, Supporting Information). After establishing that BMG can efficiently functionalize PEO with BAPy, we designed a study to verify if the macroradical had a preferred reactivity site in BAPy. PEO 100 kDa was milled with either pyrene (Py) or 1‐(acetyl)pyrene (APy) (**Scheme** **2**). Interestingly, results indicate that BMG yields functionalization with both molecules, although to a smaller extent (**Table** **2**, entries 1–3), indicating that the macroradical reaction is easier than what we originally expected.

**Table 2:** BMG of PEO 100 kDa with Py, APy or BAPy.^a)^

Entry	Fluorescent probe	Initial Mw [kDa]	Final Mw [kDa]	Chains functionalized [%]^b)^
1	Py	100	23.4 ± 3.5	7.7 ± 0.9
2	APy	100	27.0 ± 2.4	11.0 ± 1.8
3	BAPy	100	25.7 ± 4.0	36.2 ± 5.2

^a)^ The polymers were precooled to −196 °C using liquid nitrogen flow, then ball milled for three cycles of: 10 min milling at 28 Hz and 2 min cooling at 5 Hz. The presented values are an average of three independent BMG experiments;

^b)^ (%) chains functionalized were calculated from the absorption of Py at 345 nm or for APy, BAPy at 356 nm, as previously described in the literature.^[^ ^34^ ^]^

**Figure 2:**
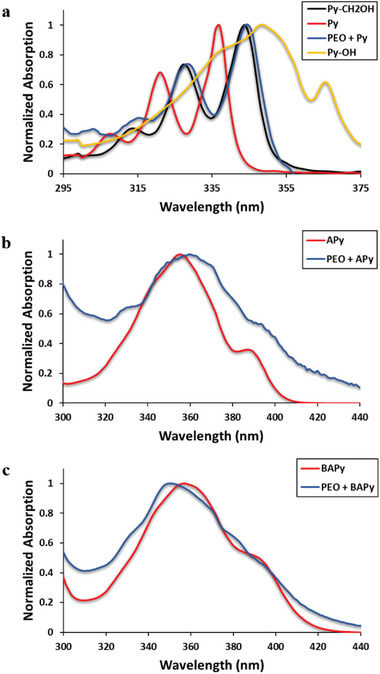
Overlay of the UV spectrum retrieved from the GPC's PDA detector with the spectrum of the adequate small molecule measured in solution. a) PEO 100 kDa after BMG with Py (blue), Py (red), Py‐CH2OH (black), and PyOH (yellow); b) PEO 100 kDa after BMG with APy (blue), APy (red); c) PEO 100 kDa after BMG with BAPy (blue), BAPy (red).

The UV absorption spectra of all three polymeric products were retrieved from the photodiode‐array (PDA) detector in the GPC, which separated the polymer from the remaining small molecule reactant. The spectra were also compared to those of the small molecules, which were measured in a spectrophotometer. When PEO is milled with Py, the resulting polymer presents a redshift adsorption relative to Py (**Figure** **2**a). Since the macroradical can only bind directly to the pyrene skeleton, a radical addition explains the change in the spectrum. Interestingly, the red‐shifted peak of the milled polymer fits nicely with the spectrum of 1‐pyrenemethanol (PyCH_2_OH), rather than the spectrum of 1‐hydroxypyrene (PyOH), suggesting the polymer reacted with Py through a carbon rather than an oxygen atom (Figure 2a). On the other hand, the spectra of the polymers that were milled with APy and BAPy overlap well with the spectra of the corresponding small molecules, APy or BAPy (Figure 2b,c), indicating that the polymer favors reacting distal to the pyrene group (and therefore in the acetyl group).

**Scheme 3:**
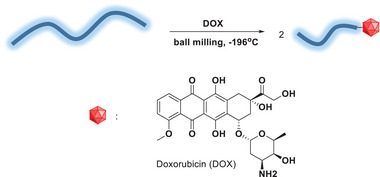
BMG of PEO 100 kDa with DOX at 28 Hz.

Given the success of this protocol in functionalizing polymers with molecules containing aromatic rings and ketones, we gathered that such a protocol could be useful for the preparation of drug‐polymer conjugates.^[^ ^35^ ^]^ Drug PEGylation is a useful method for improving the pharmacokinetic properties of drugs, i.e., increasing its half‐life in the blood stream, inhibiting its degradation by metabolic enzymes or elimination by the immune system, as well as improving its water solubility.^[^ ^36^, ^37^, ^38^ ^]^ Such a process is particularly important to hydrophobic drugs like Doxorubicin (DOX),^[^ ^39^ ^]^ a well‐known chemotherapeutic drug (**Scheme** **3**). For this purpose, PEO 100 kDa was ground in the presence of DOX following the same protocol. The solid was then analyzed using the GPC, in which again, the UV–vis PDA detector indicated the appearance of a polymer peak with a strong absorption (as opposed to the original PEO). Integrating the GPC peak at 286 nm allowed us to determine the percentage of functionalization with DOX to be 8.51 ± 0.66%. The functionalized polymer was purified by dialysis and its fluorescence spectrum was measured (excitation at 500 nm), which overlapped quite well with DOX's spectrum, providing additional support for the successful functionalization with the drug (see Figure S6, Supporting Information). Although this direct conjugation of the drug to the PEO was successful and could lead to better pharmacokinetics, a release systems would be required between the molecule and the polymer to allow such conjugates to be used for drug delivery.

**Scheme 4:**
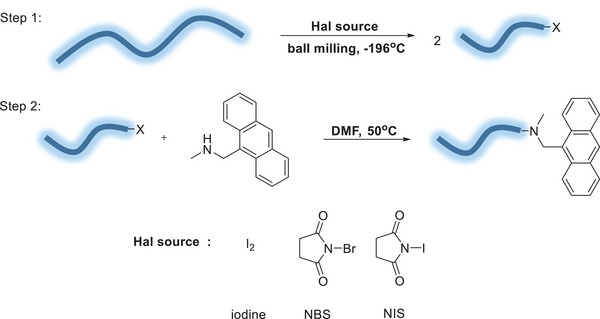
BMG of PEO 100 kDa with halogenating agent at 28 Hz, followed by substitution with 9‐(methylaminomethyl)anthracene.

While the functionalization with pyrene groups indicated macroradical reaction could be achieved in reasonable yields, and provided a good method to quantify such functionalization, these pyrene end groups are not very functional. Therefore, to make this mechanochemical C─C bond activation into more valuable end‐functionalized polymers, we targeted a different radical functionalization. Halogens are commonly installed in mono‐telechelic polymers as they can be later substituted with a nucleophile of choice to obtain large possibilities of end groups.^[^ ^40^, ^41^ ^]^ Iodine, N‐bromosuccinimide (NBS) and N‐iodosucciminde (NIS) are known radical‐halogenating agents, and could be adequate for this functionalization protocol. The reaction with PEO 100 kDa was chosen as a proof of concept for the optimization of the process. Thus, PEO was ball‐milled in the presence of each of the three halogenating agents. To quantify functionalization, after milling, the polymer was reacted in solution with 9‐(methylaminomethyl)anthracene as a nucleophile with a strong characteristic absorbance that could easily be traced using the GPC's PDA detector (**Scheme** **4**). Following this protocol for functionalization with an anthracene group, the characteristic UV spectrum was seen for the polymer peak eluted by the GPC. This indicated that the halogenation reaction and further functionalization of the polymer chains were successful (**Table** **3**). Unfortunately, the functionalization percentage calculated was smaller compared to the one obtained with BAPy. Importantly, no correlation was found between thermodynamic parameters, such as bond‐dissociation energy in the reactants, and the functionalization yield, indicating that mixing parameters are a dominating factor.^[^ ^42^ ^]^ This suggests that the bromoacetyl group in BAPy presents better reactivity to the macroradical in BMG compared to the three halogenating agents and can serve as a more adequate base for mechanochemical C─C functionalization.

**Table 3:** BMG of PEO 100 kDa with halogenating agent I_2_, NBS, NIS.^a)^

Entry	Halogenating agent	Initial Mw [kDa]	Final Mw [kDa]	Chains functionalized [%]^b)^
1	Iodine	100	19.7 ± 4.3	15.8 ± 4.4
2	NBS	100	17.4 ± 6.8	19.0 ± 0.7
3	NIS	100	21.8 ± 3.8	10.4 ± 2.6

^a)^ The polymers were precooled to −196 °C using liquid nitrogen flow, then ball milled for three cycles of: 10 min milling at 28 Hz and 2 min cooling at 5 Hz. The values presented are an average of three independent BMG experiments;

^b)^ (%) chains functionalized was calculated from the absorption of 9‐(methylaminomethyl)anthracene at 370 nm, as previously described in the literature.^[^ ^34^ ^]^

**Scheme 5:**
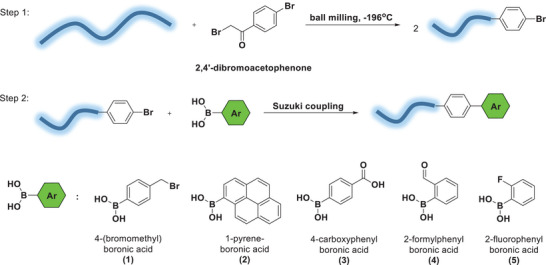
BMG of PEO 100 kDa with 2,4′‐dibromoacetophenone at 28 Hz, followed by a Suzuki reaction with various aryl boronic acid derivatives.

Therefore, in a trial to achieve similar end‐chain functionality, we considered repeating the protocol with a molecule similar to BAPy in which the aryl is brominated, then such halogen could serve as a selective site for Suzuki coupling with any arylated boronic acid. Therefore, PEO was milled in the presence of commercially available 2,4′‐dibromoacetophenone. This molecule was chosen because it's easily available and contains both the macroradical attachment point (bromoacetyl moiety), and an aryl bromide. Again, using the PDA detector in the GPC, we were able to confirm that PEO was functionalized with the small molecule, indicated by the appearance of a peak at 270 nm (see Figure S7, Supporting Information). However, the percentage functionalization with 2,4′‐dibromoacetophenone was found to be only ca. 8%. To confirm the functionalization and functionality, this aryl‐bromide functionalized PEO was reacted in solution with five different boronic acid derivatives in a Suzuki‐type coupling (**Scheme** **5**). The corresponding functionalized polymers were injected into the GPC and their UV absorption was compared to the parent polymer before the coupling step. In each case, the UV spectra showed a red shift providing different absorption patterns, and supporting the success of the coupling step (see Figure S7, Supporting Information).

**Scheme 6:**
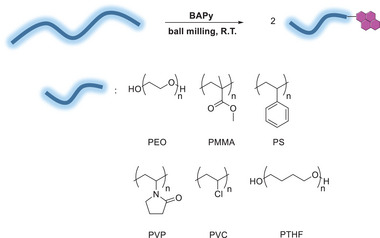
BMG of BAPy at 28 Hz with different polymers PMMA, PS, PVP, PVC, and PTHF.

Finally, to check if this BMG protocol is general, additional polymers were grinded in the presence of BAPy: polymethylmethacrylate (PMMA), polystyrene (PS), polyvinylpyrrolidone (PVP), polyvinyl chloride (PVC) and polytetrahydrofuran (PTHF) (**Scheme** **6**). All polymers showed meaningful functionalization with the BAPy group (**Table** **4**), supporting that this approach is suitable for different polymer chemistries. BMG, in this case, was done at room temperature, showing a more realistic protocol could also work for mechanochemical C─C activation. PTHF was the only polymer chemistry tested that actually required cryo‐milling, given its very low glass‐transition temperature (−80 °C).^[^ ^43^ ^]^

**Table 4:** BMG of PEO, PMMA, PS, PVP, PVC, and PTHF with BAPy.^a)^

Entry	Polymer	Initial Mw [kDa]	Final Mw [kDa]	Chains functionalized [%]^b)^
1	PEO	100.0	58.4 ± 5.8	42.9 ± 3.2
2	PMMA	618.0	24.0 ± 4.1	30.0 ± 1.8
3	PS	192.0	18.8 ± 1.7	17.8 ± 1.0
4	PVP	360.0	28.0 ± 1.9	73.0 ± 4.7
5	PVC	233.0	117.8 ± 15.0	64.5 ± 0.5
6^c)^	PTHF	77.4	51.5 ± 7.0	11.4 ± 0.9

^a)^ The polymers were ball‐milled for 30 min at 28 Hz. The values presented are an average of three independent BMG experiments;

^b)^ (%) chains functionalized were calculated from the absorption of BAPy at 356 nm as described previously in the literature^[^ ^34^ ^]^;

^c)^ The polymer was precooled to −196 °C using liquid nitrogen flow, then ball milled for three cycles of: 10 min milling at 28 Hz and 2 min cooling at 5 Hz.

## 3 Conclusion

To conclude, we showed that the mechanochemical homolytic C─C bond scission in homopolymers can be utilized to introduce new functional groups to the formed macroradicals yielding end‐functionalized polymer chains, effectively carrying out an unstrained C─C bond activation and functionalization. BMG of PEO in the presence of BAPy yielded the pyrene terminated polymer chain as evidenced by the characteristic UV absorption spectrum observed by the GPC's PDA detector, reaching up to 70% chain functionalization. Ball milling of PEOs with different molecular weights indicate that the reaction is mechanochemical in nature, as functionalization was proportional to initial molecular weight and polymers under 20 kDa were not functionalized. Comparing the reaction with BAPy, APy, and Py showed that the bromoacetyl moiety is a good site to achieve more substantial functionalization. Furthermore, we demonstrate that this protocol can be used for small drug PEGylation, demonstrated with doxorubicin. In addition, halogen end group functionalization, although in lower yields compared to BAPy, was also demonstrated by BMG of PEO with three different halogenation agents: iodine, NBS, and NIS. The halogenated polymer was reacted with an amine substituted anthracene, to quantify the amount of halogenation. Alternatively, we showed that an aryl‐bromide moiety can be installed by grinding PEO in the presence 2,4′‐dibromoacetophenone. This end group was coupled to different aryl boronic acids via Suzuki coupling, demonstrating that the product polymers are indeed semi‐telechelic. Finally, we showed that this protocol can also be applied to other carbon‐based polymers (PMMA, PS, PVP, PVC, and PTHF), suggesting it is suitable to functionalize a variety of polymer chemistries.

## 4 Experimental Section

### Synthesis—Preparation of 1‐(bromoacetyl)pyrene (BAPy)

1‐(bromoacetyl)pyrene was synthesized according to a reported procedure.^[^ ^44^ ^]^


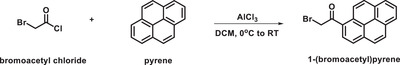


Aluminum trichloride (1.18 g, 8.85 mmol) was suspended in dichloromethane (6 mL) under argon atmosphere, then cooled to 0 °C. 2‐bromoacetyl chloride (0.65 mL, 7.80 mmol) was added dropwise, followed by pyrene (1.50 g, 7.42 mmol). After the addition was finished, the reaction's color turned red. It was allowed to warm to room temperature and stirred overnight. At this point, a precipitate was observed. The mixture was diluted with dichloromethane (200 mL) and washed twice with H_2_O (150 mL), and then with brine (150 mL). The organic layer was separated, dried over magnesium sulfate, filtered, and concentrated to yield the crude product as a yellow‐brownish solid. The crude product was purified by SiO_2_ column chromatography using ethyl acetate/hexane (v/v, 75/15) as eluent, then re‐crystalized in dichloromethane/heptane to yield the pure product as a yellow‐orange solid (0.73 g, 30% yield).

^1^H NMR (400 MHz, CDCl_3_ δ): 8.98 (d, *J* = 9.4 Hz, 1H), 8.36 (d, *J* = 8.1 Hz, 1H), 8.27 (dd, *J* = 11.1, 7.0 Hz, 3H), 8.19 (t, *J* = 8.9 Hz, 2H), 8.12–8.04 (m, 2H), 4.73 (s, 2H).

^13^C NMR (400 MHz, CDCl_3_ δ): 194.6, 134.6, 131.0, 130.6, 130.4, 130.3, 130.2, 128.4, 127.0, 126.8, 126.7, 126.6, 126.5, 125.1, 124.6, 124.1, 123.9, 34.2.

### Synthesis—Synthesis of poly(tetrahydrofuran)

Poly(tetrahydrofuran) was synthesized according to a reported procedure.^[^ ^45^ ^]^


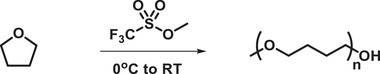


THF (100 mL, without BHT) was cooled to 0 °C under an argon atmosphere. Methyl triflate (100 µL, 0.9 mmol) was added by syringe. The reaction was allowed to warm and stirred at room temperature for 14 h. Then, the viscous solution was diluted by adding THF and the polymer was precipitated in water, which was dried in high vacuum, yielding PTHF as a white solid (46.2 g, 52% yield).

^1^H NMR (400 MHz, CDCl_3_ δ): 3.34 (m, 1H), 1.55 (m, 1H). GPC (THF), Mn = 61.3 kDa, Mw = 77.4 kDa, *Đ*  = 1.26.

### Synthesis—Procedure for the Suzuki coupling of arylboronic acids to the aryl‐bromide functionalized PEO

To a Schlenk flask aryl‐bromide functionalized PEO (10 mg), tetrakis(triphenylphosphine)palladium (Pd(PPh_3_)_4_) (11 mg, 9 µmol), K_2_CO_3_ (50 mg, 0.36 mmol) and 4‐(bromomethyl)phenylboronic acid/pyrene‐1‐boronic acid/4‐carboxyphenylboronic acid/2‐formylphenylboronic acid/2‐fluorophenylboronic acid (91 µmol) were added. The flask was flushed with argon, then water (1 mL) was added followed by 1,4‐dioxane (0.5 mL). The reaction was freeze‐pump thawed three times, backfilled with argon then heated to 70 °C overnight. After the reaction completion, the reaction was cooled to RT and the solvent evaporated. A sample was dissolved in DMF, filtered, and injected to the GPC for analysis.

### BMG Experiments—Procedure for the functionalization of PEO with pyrene/1‐(acetyl)pyrene/1‐(bromoacetyl)pyrene/2,4′‐dibromoacetophenone/doxorubicin

PEO (100 mg) and pyrene/1‐(acetyl)pyrene/1‐(bromoacetyl)pyrene/2,4′‐dibromoacetophenone/doxorubicin (34 µmol) were dry mixed using a pestle, and then transferred to a stainless‐steel milling jar (10 mL) with two stainless‐steel balls (7 mm, diameter). The jar was sealed and placed in the ball mill. The ball mill jar was precooled to −196 °C for 7 min using liquid nitrogen flow, then ball milled for three cycles of 10 min milling at 28 Hz and 2 min cooling at 5 Hz. After the milling was complete, a sample was dissolved in DMF and injected into the GPC for analysis.

### BMG Experiments—Procedure for the functionalization of PMMA/PS/PVC/PVP with 1‐(bromoacetyl)pyrene

PMMA/PS/PVC/PVP (100 mg) and 1‐(bromoacetyl)pyrene (34 µmol) were dry mixed using a pestle, then transferred to a stainless‐steel milling jar (10 mL) with two stainless‐steel balls (7 mm, diameter). The jar was sealed and placed in the ball mill, then milled for 30 min at 28 Hz. After the milling was completed, a sample was dissolved in DMF and injected to the GPC for analysis.

### BMG Experiments—Procedure for the functionalization of PTHF with 1‐(bromoacetyl)pyrene

PTHF (100 mg) and 1‐(bromoacetyl)pyrene (34 µmol) were transferred to a stainless‐steel milling jar (10 mL) with two stainless‐steel balls (7 mm, diameter). The jar was sealed and placed in the ball mill. The ball mill jar was precooled to −196°C for 7 min using liquid nitrogen flow, then ball milled for three cycles of 10 min milling at 28 Hz and 2 min cooling at 5 Hz. After the milling was complete, a sample was dissolved in THF and injected to the GPC for analysis.

### BMG Experiments—Procedure for the mechanochemical radical halogenation of PEO (PEO‐X)

PEO (100 mg) and iodine/N‐bromosuccinimide/N‐iodosuccinimide (34 µmol) were dry mixed using a pestle, then transferred to a stainless‐steel milling jar (10 mL) with two stainless‐steel balls (7 mm, diameter). The jar was sealed and placed in the ball mill. The ball mill jar was precooled to −196°C for 7 min using liquid nitrogen flow, then ball milled for three cycles of 10 min milling at 28 Hz and 2 min cooling at 5 Hz. After the milling was completed, a sample was dissolved in DMF and injected into the GPC for analysis.

### BMG Experiments—Functionalization of the halogenated PEO with 9‐(methylaminomethyl)anthracene

PEO‐X (5 mg) and 9‐(methylaminomethyl)anthracene (10 µmol) were dissolved in DMF (0.5 mL), then heated to 50 ˚C overnight. A sample was diluted in DMF to a concentration of 5 mg ml^−1^ and injected into the GPC for analysis.

### Calculation of Chain Functionalization

Chain functionalization was calculated directly from the UV–vis detector of the GPC, as described previously in the literature.^[^ ^34^ ^]^ For more details, see Section SIII (Supporting Information).

## Conflict of Interest

The authors declare no conflict of interest.

## Supporting information

Supporting Information

## Data Availability

The data that support the findings of this study are available in the supplementary material of this article.
