# Supplementary material for: Semi‐Telechelic Polymers from Mechanochemical C─C Bond Activation
Source: Adv Sci (Weinh). 2023 Oct 23;10(34):2304571. doi: 10.1002/advs.202304571 (PMC10700232; doi:10.1002/advs.202304571)
Supplement: Supplementary file 1 — Supporting Information [file ADVS-10-2304571-s001.pdf]

ADVANCED SCIENCE
Open Access

Supporting Information

for *Adv. Sci.*, DOI 10.1002/advs.202304571

Semi-Telechelic Polymers from Mechanochemical C—C Bond Activation

*Rony Schwarz and Charles E. Diesendruck**

Supporting Information

Semi-telechelic Polymers from Mechanochemical C-C Bond Activation

*Rony Schwarz and Charles E. Diesendruck**

Supporting Information

Table of Contents

I. Instrumentation and Chemicals..S3

II. Characterization and Results..S4

III. Calculation of Chain Functionalization..S10

IV. CoGEF…………………………………………………………………..………….S11

V. References...S12

I. Instrumentation and Chemicals

All chemicals and reagents were purchased from commercial sources and used without further purification, unless specified. Poly(ethylene glycol)/poly(ethylene oxide) (PEG/PEO) 1.5, 20, 100, and 400 kDa; polystyrene (PS); poly(vinyl chloride) (PVC); and poly(vinylpyrrolidone) (PVP) were purchased from Sigma-Aldrich. Poly(ethylene glycol) 4 kDa and poly(methylmethacrylate) (PMMA) were purchased from Alfa Aesar. Gel permeation chromatography (GPC) analyses were performed in dimethylformamide (DMF) containing 0.02M of LiBr (50°C, at a flow rate of 0.5 mL/min) using a Thermo LC system equipped with two TSKgel G4000HHR columns in sequence; or in THF (30°C, at a flow rate of 1.0 mL/min) using a Thermo LC system equipped with Tosoh's TSKgel Guard Column HHR-L and four TSKgel G4000HHR columns in sequence. Detection was carried out with a "triple-detector" system which includes a Dionex DAD-3000 PDI UV-Vis Detector, Wyatt OPTILAB T-rEX refractometer, and a Wyatt MALS DAWN HELEOS II 8+TR. Wyatt's Astra 7.1.4 software was used for GPC data analysis and polymer properties calculation (molecular weights, polydispersities, etc). To calculate the molecular weight for PEO functionalized with doxorubicin (DOX), polystyrene standard samples (Agilent EasiVial GPC/SEC calibration standards) were used for molecular weight standard calibration as DOX absorbs light at the MALS laser wavelength. UV-Vis was measured in a Thermo Evolution 220 Spectrophotometer. Fluorescence was measured using a fluorescence spectrometer (HORIBA Jobin Yvon Fluorolog-3). Spectra/Por® 6 dialysis membranes MWCO 1 kDa were used for dialysis. Ball milling was performed using a Retsch Cryomill with or without cooling with liquid nitrogen, using stainless steel jar and balls. All NMR spectra were recorded using Bruker AVANCE III 400 MHz spectrometers at the Technion NMR facilities. Chemical shifts are given in ppm relative to TMS.

II. Characterization & Results

NMR spectra of BAPy

a

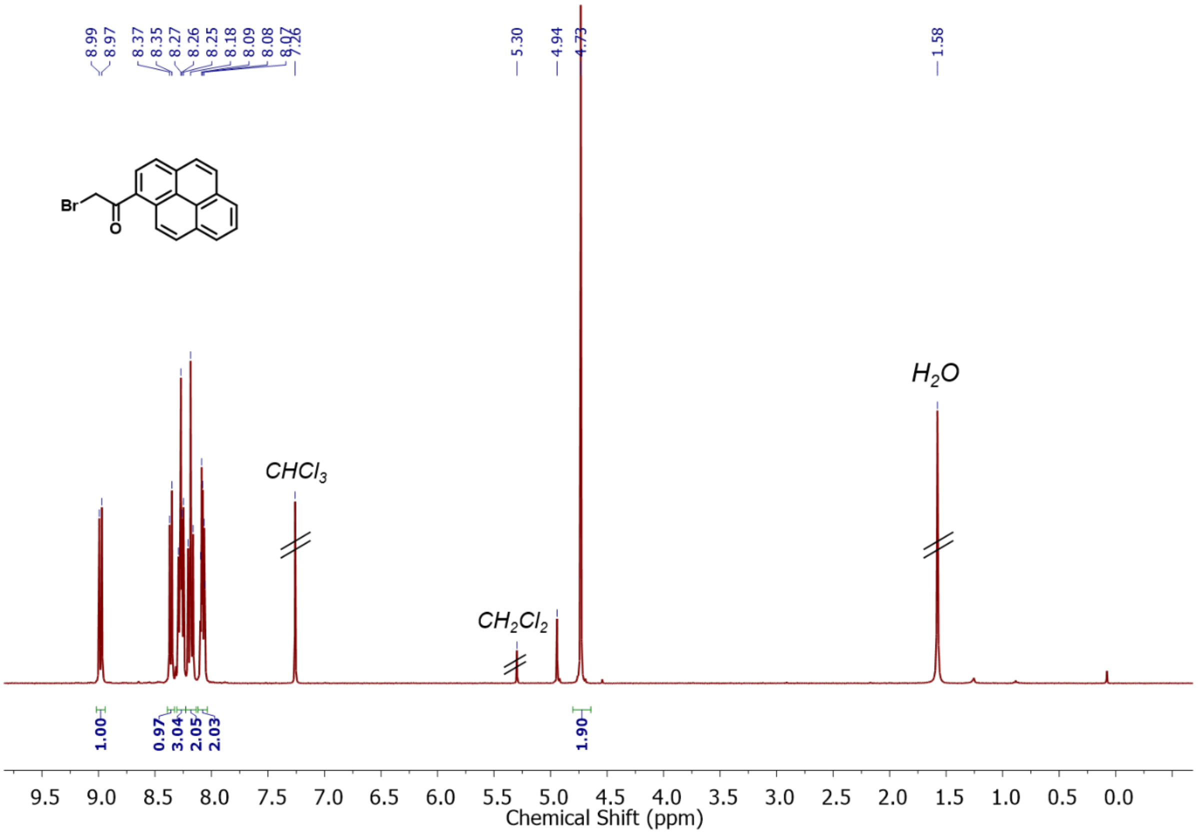

b

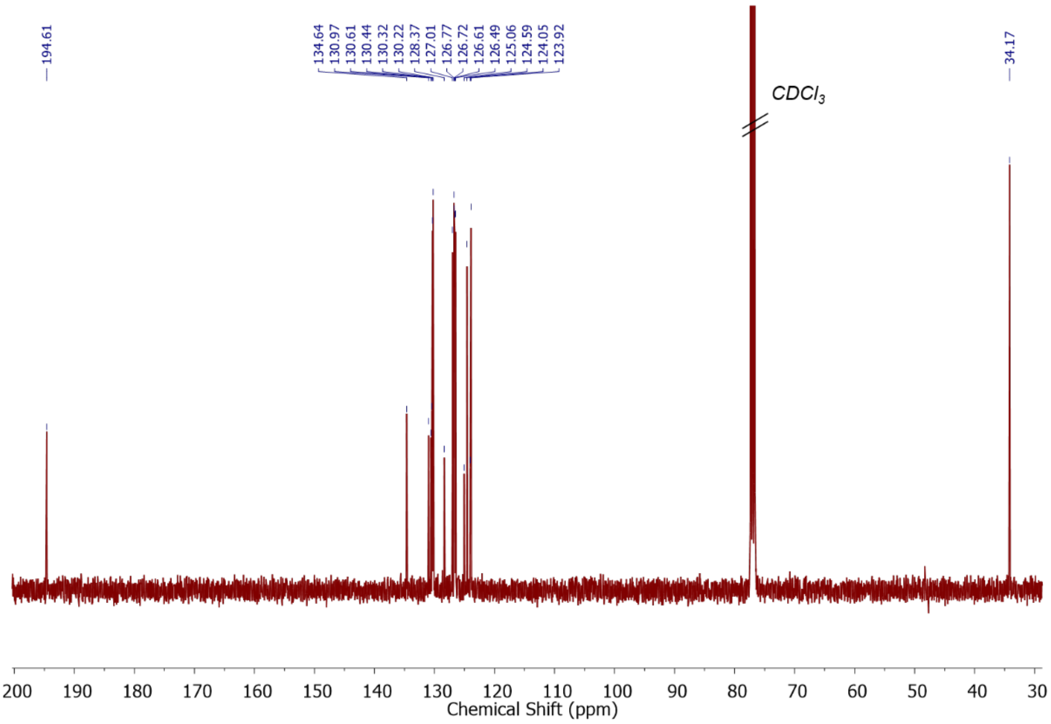

Figure S1. ^{1}H (**a**) and ^{13}C (**b**) NMR spectra of BAPy in $CDCl_3$

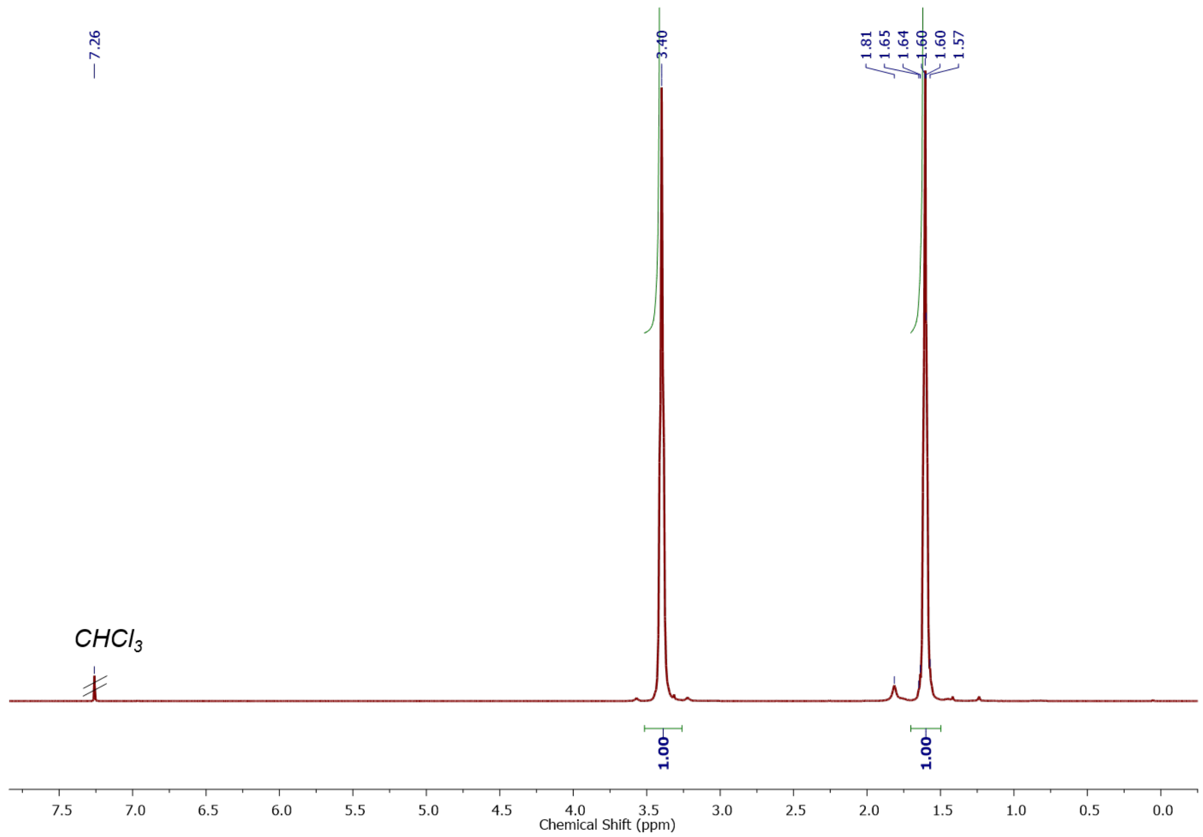

Figure S2. ^{1}H NMR spectrum of poly(tetrahydrofuran) in $CDCl_3$

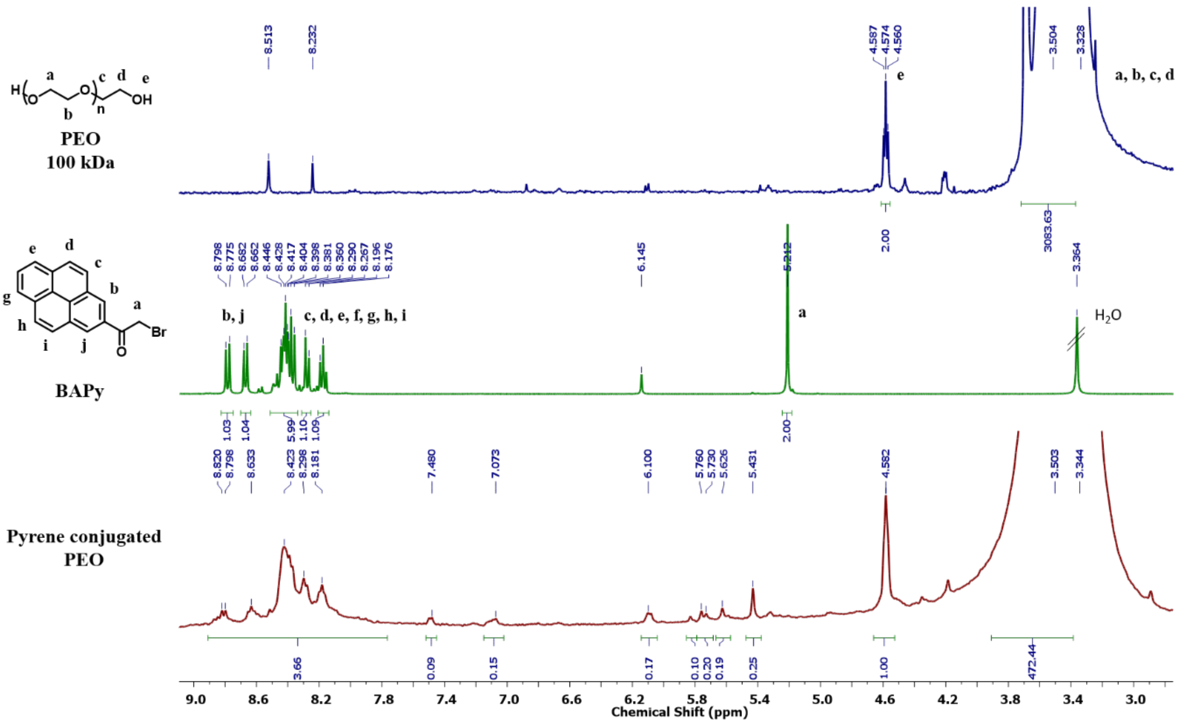

Figure S3. ^{1}H NMR spectrum of PEO 100 kDa, BAPy and the PEO after BMG with BAPy (after 6 milling cycles) in DMSO-d^6.

Chromatograms

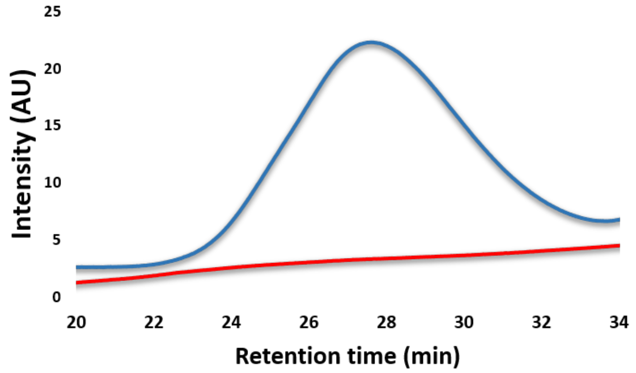

Figure S4. GPC chromatograms as observed by the UV detector at 275 nm of PEO 100 kDa (red) and PEO after BMG with doxorubicin (blue).

UV-Vis spectra

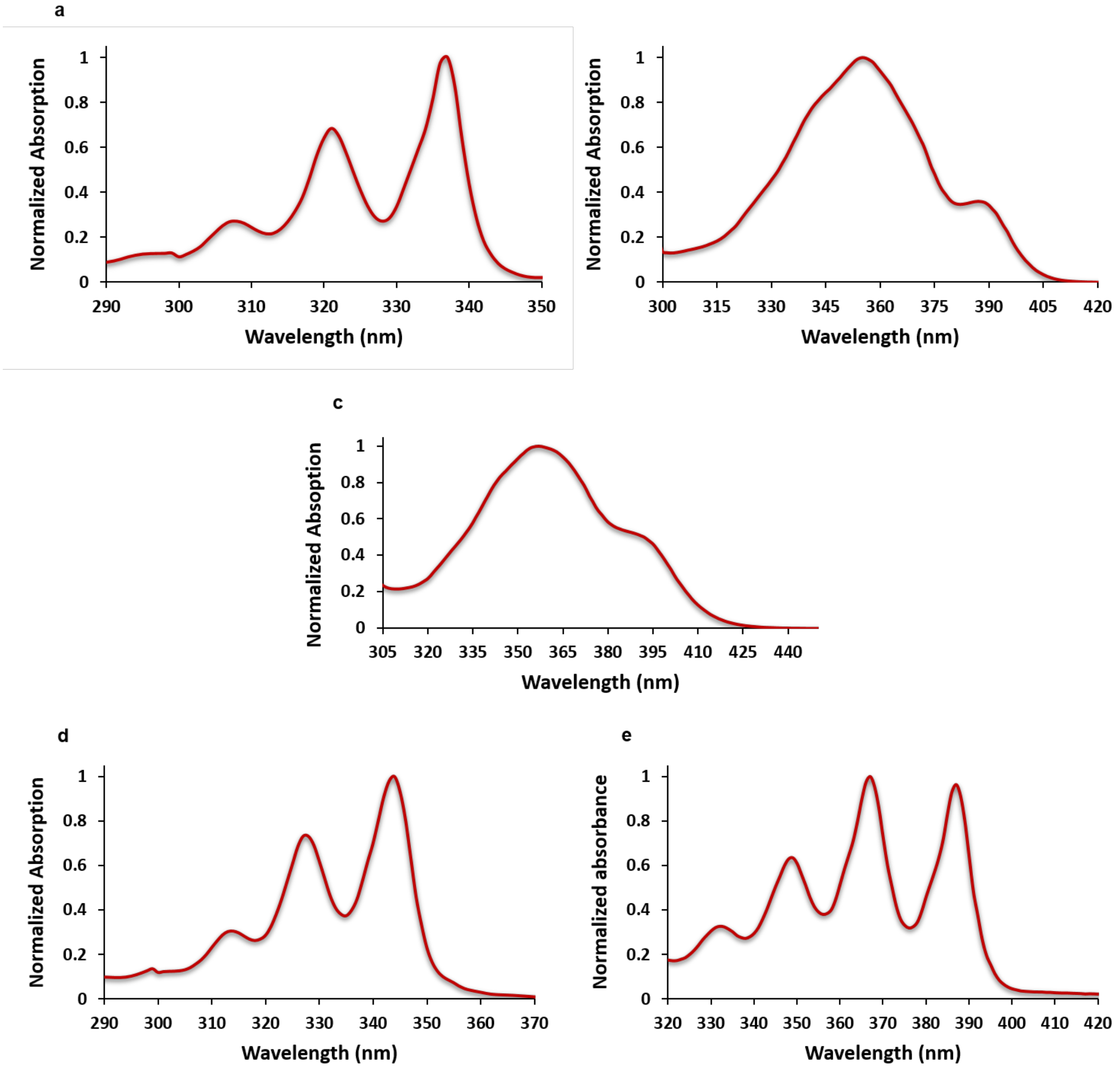

Figure S5. UV-Vis spectra of pyrene (**a**) 1-(acetyl)pyrene (**b**) 1-(bromoacetyl)pyrene (**c**) 1-pyrenemethanol (**d**) 9-(methylaminomethyl)anthracene (**e**) in DMF.

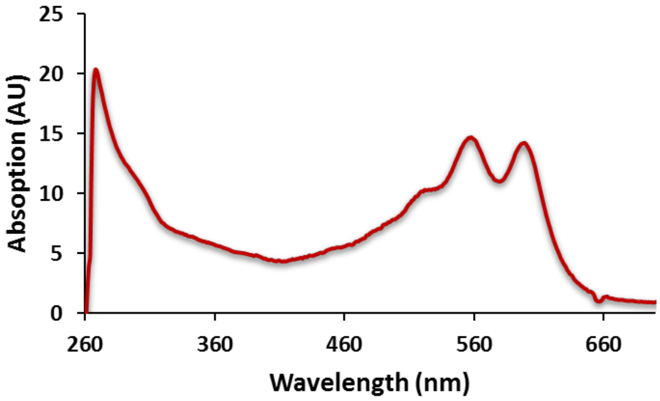

Figure S6. UV-Vis spectrum retrieved from the GPC's PDA detector of the doxorubicin functionalized PEO.

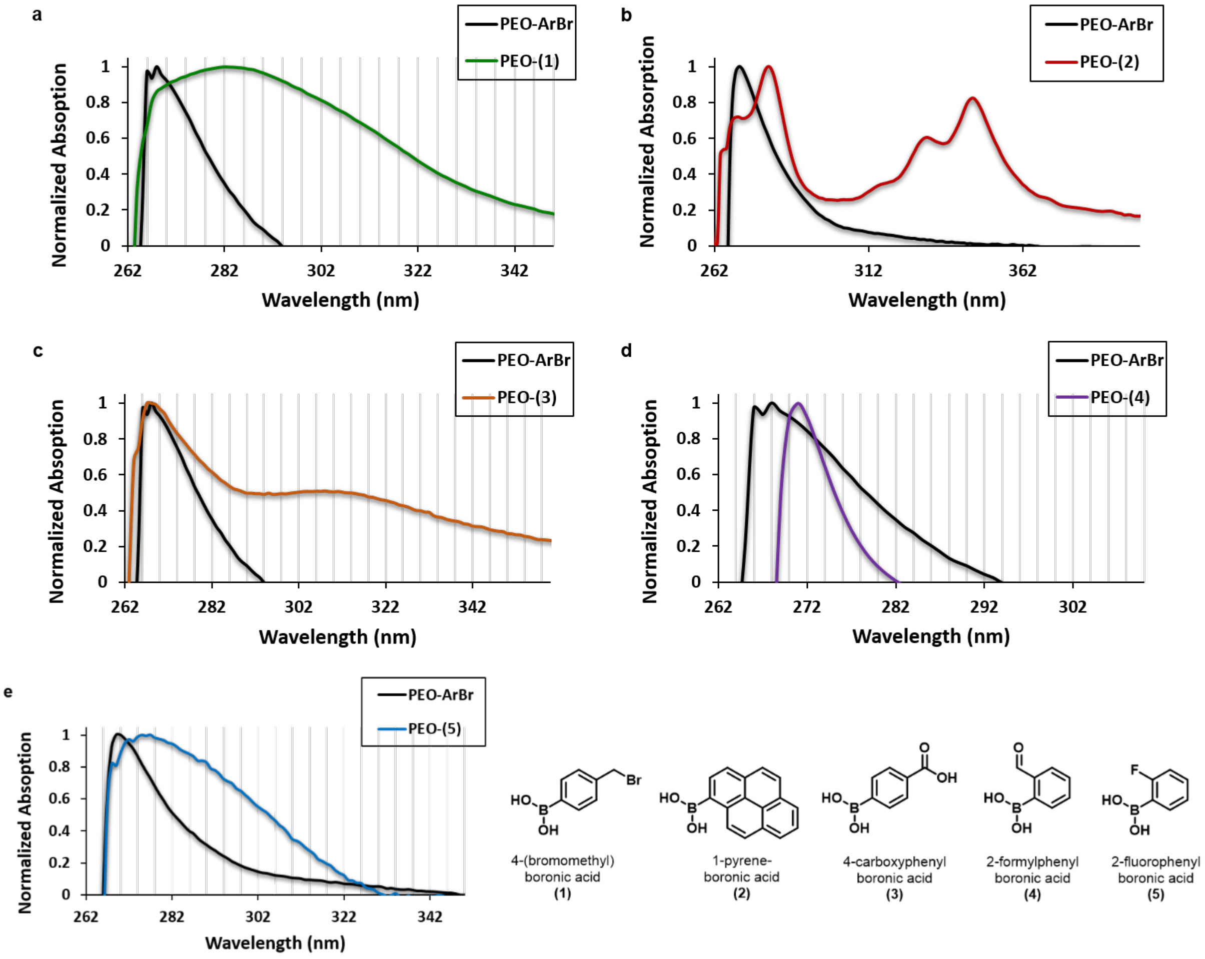

Figure S7. Overlay of the UV spectrum retrieved from the GPC's PDA detector of the aryl-bromide functionalized PEO (black) with (**a**) the functionalized PEO after the Suzuki coupling with (**1**) (green); (**b**) the functionalized PEO after the Suzuki coupling with (**2**) (red); (**c**) the functionalized PEO after the Suzuki coupling with (**3**) (orange); (**d**) the functionalized PEO after the Suzuki coupling with (**4**) (purple); and (**e**) the functionalized PEO after the Suzuki coupling with (**5**) (blue).

Fluorescence spectra

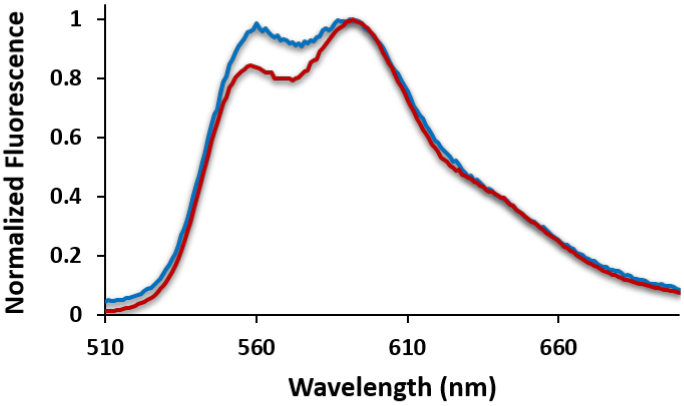

Figure S8. Fluorescence spectra (in H_2O) observed of doxorubicin (red) and doxorubicin functionalized PEO after dialysis (blue). Excitation wavelength 500 nm.

Calculations of excitation coefficients

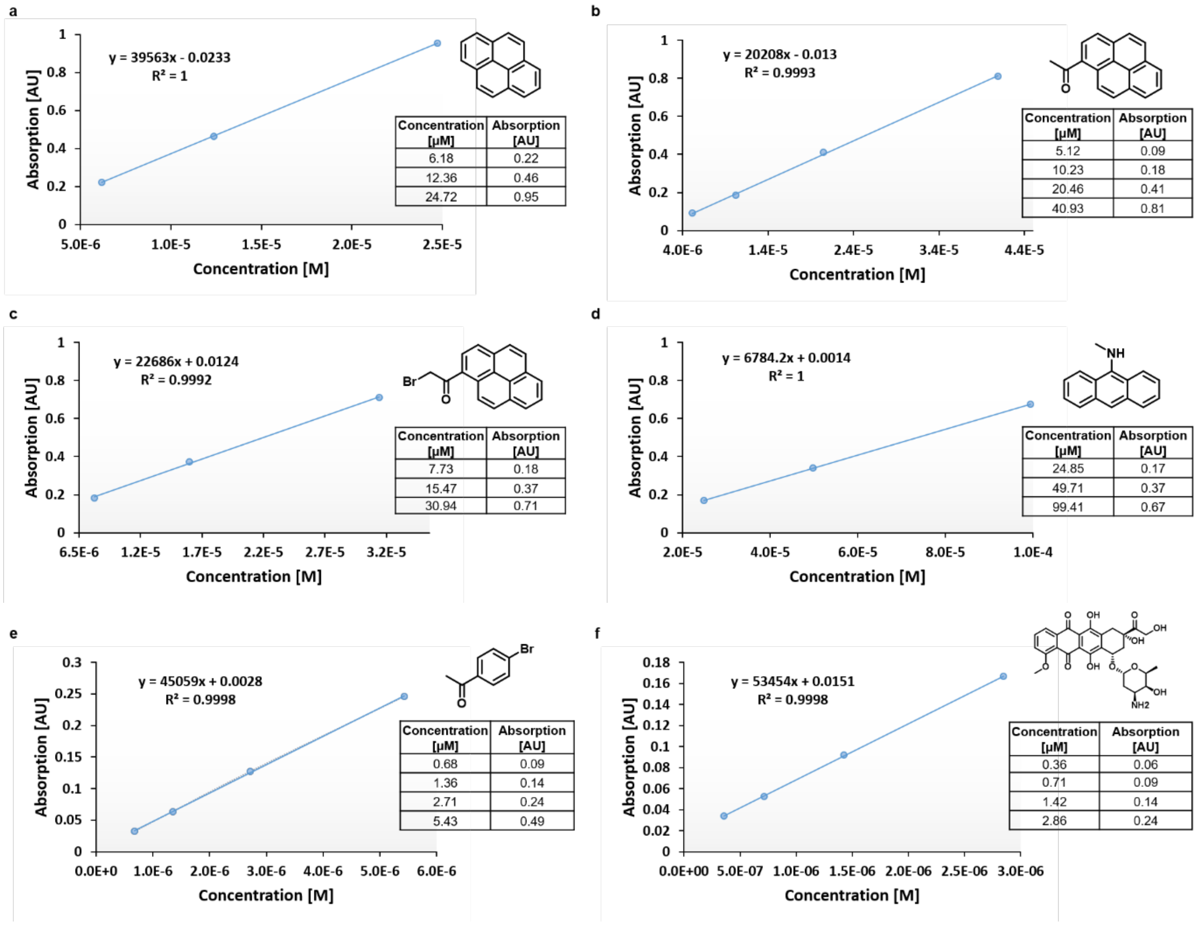

Figure S9. Calibration curves of pyrene (**a**) 1-(acetyl)pyrene; (**b**) 1-(bromoacetyl)pyrene; (**c**) 1-(bromoacetyl)pyrene; (**d**) 9-(methylaminomethyl)anthracene; (**e**) 4-bromoacetophenone; and (**f**) doxorubicin; in DMF.

Table S1. BMG of PEO of different molecular weights with BAPy.[a)]

	Initial molecular weight (Kg/mol)	V1 (min)	V2 (min)	Area at 356nm (mAU*min)	Polymer Mw (gr/mol)	Average Mw (gr/mol)	std dev	mol of pyrene (mol)	mol of polymer (mol)	Chains functionalized (%)[b)]	Average chains functionalized (%)	std dev
1		0.00	0.00	0.00	3194			0.00	0.00	0.00		
2	1.5	0.00	0.00	0.00	1034	2573	1341	0.00	0.00	0.00	0.00	0.00
3		0.00	0.00	0.00	3492			0.00	0.00	0.00		
1		0.00	0.00	0.00	3689			0.00	0.00	0.00		
2	4	0.00	0.00	0.00	5694	4935	1087	0.00	0.00	0.00	0.00	0.00
3		0.00	0.00	0.00	5421			0.00	0.00	0.00		
1		11.80	16.60	9.82	11600			6.34E-10	2.21E-08	2.87		
2	20	11.60	16.50	11.67	9947	11232	1147	7.30E-10	2.81E-08	2.59	2.71	0.15
3		11.80	16.60	12.51	12150			8.08E-10	3.03E-08	2.67		
1		10.90	16.50	59.88	30350			3.18E-09	8.96E-09	35.52		
2	100	10.90	16.70	64.22	27630	29617	1740	3.32E-09	9.55E-09	34.72	36.22	1.94
3		10.85	16.50	75.74	30870			3.98E-09	1.04E-08	38.42		
1		10.60	16.05	60.12	49640			3.19E-09	5.96E-09	53.57		
2	400	10.05	16.70	59.43	63960	62030	11547	2.58E-09	6.13E-09	42.09	45.29	7.22
3		10.15	16.80	58.88	72490			2.58E-09	6.40E-09	40.23		
1		10.65	16.80	192.83	29090			9.32E-09	1.18E-08	78.85		
2	100[c)]	10.95	17.05	133.09	27890	28770	771	6.62E-09	9.18E-09	72.17	75.57	3.34
3		10.80	16.80	165.56	29330			8.26E-09	1.09E-08	75.70		
1		10.05	17.00	75.6911	63900			3.17E-09	7.76E-09	40.89		
2	100[d)]	10.20	17.20	102.7567	52390	58400	5772	4.33E-09	9.31E-09	46.53	42.86	3.18
3		10.15	17.10	52.9088	58910			2.23E-09	5.43E-09	41.16		

a) The polymers were precooled to -196°C using liquid nitrogen flow, then ball milled for three cycles of 10 min milling at 28 Hz and 2 min cooling at 5 Hz; b) (%) chains functionalized was calculated from the absorption of BAPy at 356 nm as described at part IV; c) Six cycles were done instead of three; d) The polymer was ball milled for 30 minutes at 28 Hz at room temperature.

Table S2. BMG of PEO 100 kDa with Py, APy, BAPy, 2,4'-dibromoacetophenone or doxorubicin.[a)]

	Fluorescent probe	V1 (min)	V2 (min)	Area (mAU*min)	Polymer Mw (gr/mol)	Average Mw (gr/mol)	std dev	mol of pyrene (mol)	mol of polymer (mol)	Chains functionalized (%)[b)]	Average chains functionalized (%)	std dev
1		10.65	18.25	42.70	21130	23377	3534	1.00E-09	1.40E-08	7.15	7.70	0.92
2	Py	10.85	18.90	50.31	21550			1.15E-09	1.60E-08	7.18		
3		10.45	18.70	35.27	27450			7.66E-10	8.74E-09	8.76		
1		10.60	17.85	28.94	27100	26993	2392	1.37E-09	1.09E-08	12.58	10.97	1.85
2	APy	10.50	18.05	25.83	29330			1.18E-09	1.04E-08	11.38		
3		10.45	18.45	24.78	24550			1.08E-09	1.21E-08	8.95		
1		10.75	16.25	99.67	22040	25727	4037	5.32E-09	1.27E-08	41.85	36.18	5.23
2	BAPy	10.90	16.50	59.88	30040			3.18E-09	9.05E-09	35.15		
3		10.90	16.70	64.22	25100			3.32E-09	1.05E-08	31.54		
1	2,4'-	11.35	17.60	58.10	23960			1.47E-09	1.87E-08	7.86		
2	dibromoacet	11.60	17.40	44.08	20850	21977	1723	1.17E-09	1.69E-08	6.92	7.19	0.58
3	ophenone	11.55	17.55	62.85	21120			1.62E-09	2.39E-08	6.80		
1		11.60	16.90	32.41	53959			8.06E-10	8.90E-09	9.06		
2	Doxorubicin	11.95	17.00	46.34	45281	50062	4406	1.23E-09	1.41E-08	8.70	8.51	0.66
3		11.80	16.85	30.71	50946			8.06E-10	1.04E-08	7.78		

a) The polymers were precooled to -196°C using liquid nitrogen flow, then ball milled for three cycles of 10 min milling at 28 Hz and 2 min cooling at 5 Hz; b) (%) chains functionalized was calculated from the absorption of Py at 345 nm, absorption of APy, BAPy at 356 nm, absorption of 4-bromoacetophenone at 270 nm or absoption of doxorubicin at 287 nm, as described in part IV.

Table S3. BMG of PEO 100 kDa with halogenating agent Iodine, NBS, NIS.[a)]

	Halogenating agent	V1 (min)	V2 (min)	Area at 370nm (mAU*min)	Polymer Mw (gr/mol)	Average Mw (gr/mol)	std dev	mol of anthracene (mol)	mol of polymer (mol)	Chains functionalized (%)[b)]	Average chains functionalized (%)	std dev
1		11.35	16.65	19.93	20930			3.83E-09	1.91E-08	20.05		
2	Iodine	11.40	16.45	13.90	23240	19687	4312	2.79E-09	1.72E-08	16.23	15.83	4.43
3		11.45	16.40	14.69	14890			3.01E-09	2.69E-08	11.21		
1		12.15	17.65	18.97	19450			3.74E-09	2.06E-08	18.21		
2	NBS	11.50	17.35	18.80	22910	17392	6785	3.37E-09	1.75E-08	19.30	19.03	0.73
3		11.95	17.70	42.52	9817			7.98E-09	4.07E-08	19.58		
1		11.60	16.05	7.94	18240			1.80E-09	2.19E-08	8.22		
2	NIS	11.60	16.05	7.99	21220	21777	3845	1.81E-09	1.89E-08	9.62	10.35	2.58
3		11.70	16.10	8.85	25870			2.04E-09	1.55E-08	13.22		

a) The polymers were precooled to -196°C using liquid nitrogen flow, then ball milled for three cycles of 10 min milling at 28 Hz and 2 min cooling at 5 Hz; b) (%) chains functionalized was calculated from the absorption of BAPy at 356 nm as described in part IV.

Table S4. BMG of BAPy at 28 Hz with different polymers PMMA, PST, PVP, PVC and PTHF.[a)]

	Polymer	V1 (min)	V2 (min)	Area at 356nm (mAU*min)	Polymer Mw (gr/mol)	Average Mw (gr/mol)	std dev	mol of pyrene (mol)	mol of polymer (mol)	Chains functionalized (%)[b)]	Average chains functionalized (%)	std dev
1		12.65	17.5	35.21	28160			2.39E-09	7.95E-09	30.06		
2	PMMA	12.65	17.55	58.37	20030	23983	4070	3.93E-09	1.40E-08	28.11	30.00	1.85
3		12.50	17.55	54.43	23760			3.54E-09	1.11E-08	31.82		
1		12.20	17.65	58.20	17370			3.47E-09	1.93E-08	17.96		
2	PS	12.35	17.45	36.53	19770	18807	1268	2.33E-09	1.46E-08	15.99	17.02	0.99
3		12.35	17.30	36.93	19280			2.41E-09	1.41E-08	17.12		
1		13.20	18.35	163.57	29750			1.09E-08	1.40E-08	78.27		
2	PVP	13.20	18.25	126.75	28060	27960	1842	8.62E-09	1.20E-08	72.02	72.99	4.86
3		13.30	18.30	143.46	26070			9.91E-09	1.44E-08	68.69		
1		15.80	36.40	24.88	105800			1.31E-09	2.04E-09	64.36		
2	PVC	16.00	36.50	42.24	113000	117800	14988	2.26E-09	3.47E-09	65.08	64.55	0.46
3		19.00	35.20	17.62	134600			1.26E-09	1.96E-09	64.22		
1		23.10	37.60	13.52	43530			1.22E-09	1.07E-08	11.48		
2	PTHF	22.80	37.30	8.11	56615	51512	7001	7.26E-10	6.92E-09	10.49	11.44	0.93
3		23.10	37.20	9.23	54390			8.54E-10	6.91E-09	12.35		

a) PMMA, PS, PVP and PVC were ball milled for 30 min at 28 Hz. PTHF was precooled to -196°C using liquid nitrogen flow, then ball milled for three cycles of 10 min milling at 28 Hz and 2 min cooling at 5 Hz; b) (%) chains functionalized was calculated from the absorption of BAPy at 356 nm as described at part IV.

III. Calculation of Chain Functionalization

Chain functionalization is calculated directly from the UV-Vis detector of the GPC, as described previously in the literature.[1] Area under the peak corresponding to the functionalized polymer is used to calculate the number of moles of polymer chains functionalized with the chromophore. Using Beer-Lambert's law, the absorption is replaced with εlC, where ε is the chromophore's excitation coefficient, l is the optic path length and C is the concentration of the functionalized polymer, V_1 and V_2 are the retention volumes at the beginning and end of the polymer peak.

Area $_{\text{under the peak}} = \int_{v_1}^{v_2} A dV = \int_{v_1}^{v_2} \varepsilon lC dV$

Assuming ε and l are constant in the concentrations measured and applying $C = \frac{n}{V}$:

$\int_{v_1}^{v_2} \varepsilon l C dV \ = \ \varepsilon l \int_{v_1}^{v_2} C dV = \ \varepsilon l \int_{v_1}^{v_2} \frac{n}{V} dV = \ \varepsilon l n \int_{v_1}^{v_2} \frac{1}{V} dV = \ \varepsilon \ln\left[\ln(v_2) - \ \ln(v_1)\right] =$
$\varepsilon \ln\left[\ln\left(\frac{v_2}{v_1}\right)\right]$

Therefore, the number of moles of the functionalized polymer can be expresses as:

$$n_{func} = \frac{Area}{\varepsilon l \cdot \ln\left(\frac{v_2}{v_1}\right)}$$

The total number of the polymer chains injected to the GPC can be calculated from the experimental values: the injected concentration C, injection volume v and the polymer's molecular weight Mw:

$$n_{tot} = \frac{C\left[\frac{mg}{mL}\right] \cdot \ v\ [mL]}{Mw}$$

Now the % functionalization can be calculated by:

$$\%\ chain\ functionalization = \frac{n_{func}}{n_{tot}} \cdot 100$$

IV. CoGEF

In silico calculation were used to simulate the effect of mechanical force on triethylene glycol as a model for PEO. The Constrained Geometries simulate External Force (CoGEF) method, developed by Beyer[2] was used (figure S10). Thus, the distance between terminal oxygens were increased in each step by 0.1 Å. In each step the energy is calculated after the geometry of the molecule is minimized with the terminal O-O distance as only constraint. These calculations were done using Spartan 14 at the B3LYP/631G* level of theory. The simulation shows the molecule is gradually stretched, until finally the stress is relieved by the homolytic scission of a C-C bond.

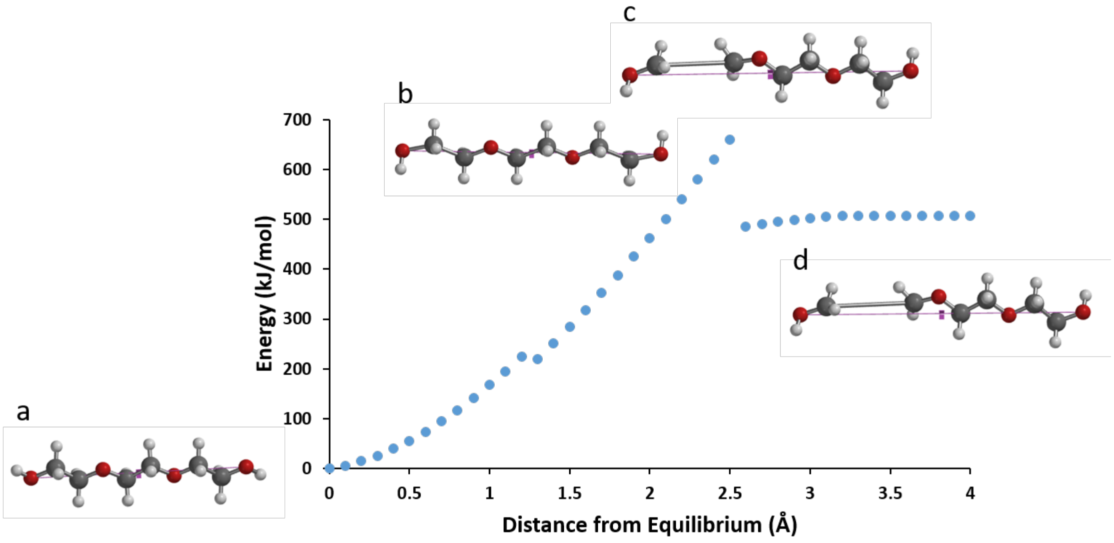

Figure S10. Plot of relative energy to relative displacement of the terminal O-O distance in triethyleneglycol, starting from an equilibrium distance between the two terminal oxygens. A stick model of the molecule at different stages of the simulation is included next to the plot: (**a**) at the minimum; (**b**) just before bond scission; (**c**) at the breaking point; and (**d**) after the C-C bond is broken.

V. References

[1] C. E. Diesendruck, L. Zhu, J. S. Moore, *Chem. Commun* **2014**, *50*, 13235.
[2] M. K. Beyer, *J. Chem. Phys.* **2000**, *112*, 7307.
